# Hi-RAGrasp: A Human-in-the-Loop Experience-Augmented Method for Task-Oriented Grasping

**DOI:** 10.3390/s26103221

**Published:** 2026-05-19

**Authors:** Yaxin Liu, Yue Hu, Yan Liu, Ming Zhong

**Affiliations:** State Key Laboratory of Robotics and System, Harbin Institute of Technology, Harbin 150001, China; liuyaxin@hit.edu.cn (Y.L.); noomi_email@163.com (Y.H.); 21b908088@stu.hit.edu.cn (Y.L.)

**Keywords:** task-oriented grasping, affordance segmentation, human-in-the-loop, retrieval-augmented generation

## Abstract

With the growing demand for assistive robots in aging societies, task-oriented grasping in household environments has become increasingly important. Compared with structured industrial settings, household scenarios are characterized by diverse objects, unstructured layouts, and strong variability in task semantics. However, traditional methods focus on geometric stability and fail to capture task-relevant semantic constraints on manipulation regions, while existing approaches suffer from unstable reasoning and lack effective mechanisms for incorporating human intervention into the reasoning process. To address these challenges, we propose Hi-RAGrasp, a task-oriented grasping framework that integrates progressive multi-stage reasoning, Human-in-the-Loop (HITL) interaction, and Retrieval-Augmented Generation (RAG). A coarse-to-fine pipeline progressively refines predictions from object-level localization to part-level grounding, enabling robust mapping from human instructions to fine-grained task-relevant regions. Meanwhile, a HITL correction mechanism and a structured human experience database are introduced and combined with RAG to form a unified paradigm that aligns with prior experience when available and falls back to reasoning otherwise, enabling experience reuse and future experience accumulation without retraining. In addition, a Geometric Heuristic Segmentation (GHS) method is proposed to improve task-relevant region localization for textureless objects. Experiments show that our method achieves a segmentation success rate of 77.73% on the evaluation dataset and a grasp success rate of 75% in real-world scenarios, significantly outperforming existing methods and demonstrating strong effectiveness and practicality in open environments.

## 1. Introduction

With the accelerating global population aging, assistive robots have emerged as a vital solution to support the daily activities of individuals with limited mobility. In domestic assistance scenarios, object grasping serves as the foundation for “fetch–use–place” task chains; its success depends not only on physical stability but also on the seamless alignment between the grasp pose and subsequent functional operations. Traditional methods often follow a task-agnostic paradigm, prioritizing geometric stability and force closure while neglecting the semantic constraints imposed by specific tasks. Unlike structured industrial settings, household environments present dual challenges: the high diversity of human instructions and the extreme variability of object instances. Consequently, achieving an accurate understanding of task semantics and generating grasping strategies that naturally facilitate subsequent operations in complex domestic settings have become critical bottlenecks in the field of assistive robotics.

The core of task-oriented grasping lies in establishing a mapping between task semantics and operational affordances, where the concept of affordance originates from Gibson’s ecological psychology [1]. These affordances are often grounded as task-relevant regions in the visual space. Recent studies on human-centered assistive manipulation further support this view. For example, mobile robot-to-human handover systems require object retrieval, grasp planning, navigation, human pose perception, and safe transfer execution to be jointly considered, indicating that grasping should be aligned with downstream human interaction requirements [2]. Similarly, dynamic tool handling with tactile myoelectric prosthetic hands shows that functional manipulation requires adaptive and stable control under varying loads and external disturbances, rather than merely maintaining a stable grasp [3]. Therefore, in robotic manipulation, this mapping requires bridging abstract human instruction and concrete pixel-level operational regions.

Early studies on task-oriented grasping primarily relied on explicit semantic modeling, constructing knowledge graphs or logical rules to characterize the relationships among tasks, objects, and manipulation parts [4,5]. While such methods exhibit strong interpretability and rule consistency within predefined semantic spaces, they are fundamentally based on a closed-world assumption, making them difficult to generalize to diverse task-object combinations in open environments. To address this limitation, subsequent work introduced demonstration-driven experience modeling, such as RTAGrasp [6] and RAM [7], which build affordance memory banks from human demonstrations and leverage retrieval mechanisms to transfer historical operational constraints to target objects. Although these approaches partially relax the rigidity of fixed semantic structures, their performance remains highly dependent on the coverage of demonstration data and can be regarded as a “weakly open” prior-driven paradigm, where generalization is strictly bounded by the diversity of the pre-recorded priors.

To further reduce annotation costs and improve scalability, recent research has shifted toward weakly supervised and self-supervised data-driven approaches. For instance, methods such as those by Luo et al. [8,9,10,11] extract shared affordance representations by modeling relational structures across different views or instances, enabling task-relevant region prediction under weak supervision. Parallel to this, another line of work leverages the cross-modal alignment capability of vision-language pre-trained models, such as CLIP [12] and DINOv2 [13], to enable open-vocabulary affordance prediction by computing feature similarities in a shared embedding space [14,15,16,17]. Despite their advantages, these approaches typically require large-scale interaction data and substantial computational resources, and their performance remains constrained by the distribution of training data. Furthermore, since alignment-based methods rely on associative matching over semantic memory, their spatial representations often lack explicit geometric topology or structural constraints, leading to blurred boundaries and instability in complex or fine-grained scenarios.

In recent years, Vision-Language Models (VLMs) have provided a new paradigm for task reasoning in open environments. Representative approaches, such as VoxPoser [18], Copa [19], AffordGrasp [20], and ReKep [21], leverage the commonsense reasoning capabilities of VLMs to interpret human instructions and ground task-relevant regions. However, the reasoning process of these methods heavily depends on pretrained knowledge, making them prone to semantic hallucinations where the model fails to align with specific physical scenes. To enhance reliability, recent studies have begun to incorporate RAG and HITL mechanisms. For instance, Affordance RAG [22] improves task performance through hierarchical retrieval, yet its modeling remains limited to object-level semantics and lacks task-relevant region grounding. Similarly, while HITL mechanisms [23] have been introduced to correct recognition errors, such corrections are primarily applied at the semantic label level and are insufficient for refining spatial representations. Critically, existing systems lack a robust mechanism to accumulate these human corrections as long-term, transferable knowledge, resulting in an open-loop inference paradigm that hinders continuous system evolution.

In summary, existing methods still face three key challenges in open environments: (1) the lack of a stable mapping between human instruction understanding and fine-grained task-relevant part grounding, making it difficult to accurately associate high-level semantics with pixel-level task-relevant regions; (2) the absence of sustainable human feedback and experience accumulation mechanisms, limiting the system’s ability to continuously improve during long-term deployment; and (3) instability in spatial representations when dealing with textureless or structurally ambiguous objects, which undermines the reliability of task-oriented grasping.

To address the aforementioned challenges, we propose a task-oriented grasping framework that integrates VLM reasoning, progressive affordance segmentation, and retrieval-augmented experience learning. The framework follows a coarse-to-fine strategy, decoupling instruction parsing, object instance segmentation, and task-relevant part grounding to establish a robust mapping from human instructions to pixel-level affordance regions. Furthermore, a HITL mechanism and a GHS module are incorporated to enable closed-loop correction and enhance spatial stability in challenging scenarios.

The main contributions of this work are summarized as follows:

1. We design a coarse-to-fine task-oriented grasping framework with a modular pipeline that progressively decomposes human instruction parsing, instance-level target object segmentation, and task-relevant part grounding, enabling a stable mapping from human instructions to executable grasp poses and improving robustness compared to one-shot reasoning approaches.
2. We propose a task-relevant region optimization method that integrates HITL and RAG. To address localization errors caused by VLM semantic reasoning bias and segmentation failures on textureless objects, HITL is introduced to correct typical erroneous results, while a GHS method provides a geometry-based segmentation pathway for textureless or structurally continuous objects. The correction information is then stored in a structured human experience database. Through a multi-level retrieval algorithm, the system retrieves relevant historical experiences and adopts a dynamic reasoning strategy: reliable experiences are used to constrain task-relevant region reasoning, while unreliable or insufficient matches trigger fallback to the default reasoning pipeline. This improves the system’s adaptability, stability, and long-term improvement capability in open household environments.
3. We conduct comprehensive evaluations on a constructed task-oriented affordance dataset and a real-world mobile manipulator platform. Through comparative experiments and ablation studies, the proposed framework demonstrates clear advantages in handling tasks with complex functional constraints and textureless objects. Real-world experiments further show that the system significantly improves task-oriented grasping success rates in multi-object cluttered scenarios, validating its reliability and practical applicability from high-level reasoning to physical execution.

## 2. Materials and Methods

**Figure 1:**
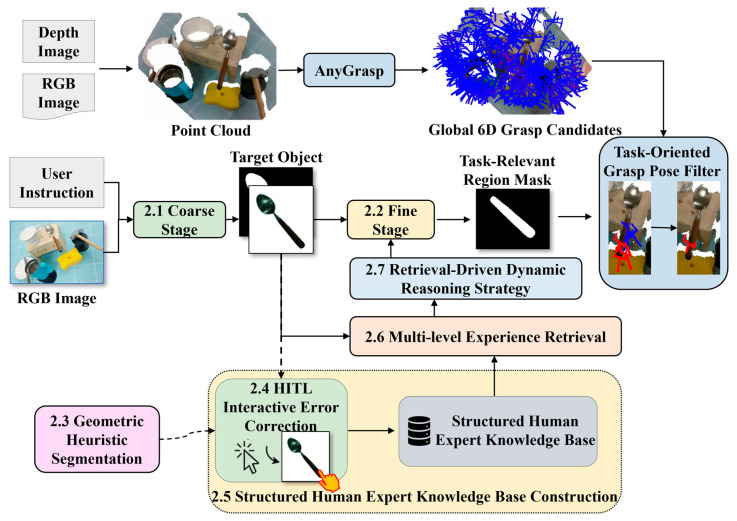
Task-Oriented Grasping Framework. The arrows indicate the workflow direction, while the dashed lines denote the components involving human-in-the-loop interaction.

This study proposes a progressive task-oriented grasping framework that integrates HITL and RAG, aiming to address the challenge of accurately identifying task-relevant object parts and guiding grasping decisions from abstract human instructions in complex household environments. As illustrated in Figure 1, the framework consists of four synergistic modules. First, an automated semantic perception pipeline leverages the collaboration of VLM and visual perception models to transform human instructions into fine-grained task-relevant regional masks through coarse-stage target localization (Section 2.1) and fine-stage part grounding (Section 2.2). Second, the correction and experience storage module introduces GHS (Section 2.3) to address segmentation failures on textureless objects, incorporates a HITL correction module (Section 2.4) for user intervention, and constructs a structured human experience database (Section 2.5) to transform correction information into reusable long-term experience. Third, an experience-driven knowledge engine employs multi-level experience retrieval (Section 2.6) and dynamic reasoning strategies (Section 2.7) to incorporate retrieved human experience during inference and adaptively adjust reasoning paths according to the retrieval results. Finally, the task-oriented grasp pose generation and filtering module (Section 2.8) maps semantic reasoning results into 3D space and executes global grasp pose filtering, ultimately achieving a complete logical closed-loop from abstract semantic understanding to task-oriented grasp pose generation.

### 2.1. Coarse Stage Target Object Localization

**Figure 2:**
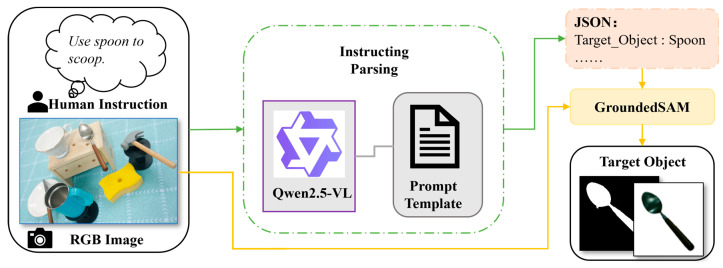
Coarse-stage target object localization framework.

In the task-oriented perception pipeline, coarse-stage target object localization transforms abstract human intent into concrete object-level spatial constraints through the synergy of human instruction parsing and instance segmentation, as illustrated in Figure 2. The process begins with the robotic perception front-end receiving the raw human instruction and the scene RGB image. The VLM is then employed to parse the human instruction and convert the unstructured natural language input into a structured JSON representation containing the task action and the target object label.

**Figure 3:**
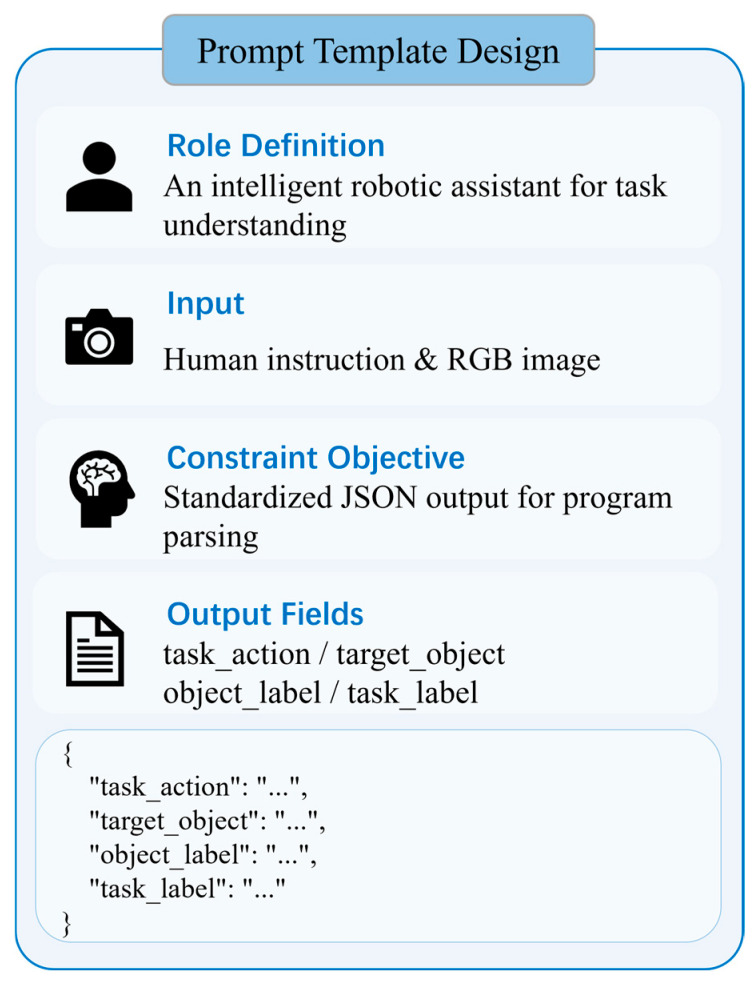
Instruction parsing prompt template.

To enable stable and consistent instruction parsing, we design a structured prompt template, as illustrated in Figure 3, to guide the VLM in generating a unified JSON representation. The target object label parsed from the instruction is then used as a textual prompt for GroundedSAM [24] to guide target object localization and mask generation. To further suppress environmental noise and emphasize the object of interest, the system applies the predicted target object mask to the original image and whitens the background, producing a background-suppressed image centered on the target object. This processed image serves as a clean input for the subsequent fine-stage task-relevant part localization. As shown in Figure 2, the spoon is successfully segmented, yielding its corresponding mask and a white-background segmentation map.

### 2.2. Fine-Stage Task-Relevant Part Grounding

Following the coarse-stage object localization described in Section 2.1, although the system has successfully localized target objects such as a “spoon,” obtaining only object-level positional information is often insufficient for supporting complex task execution. Taking a “scooping” task as an example: if the robot randomly grasps the head (bowl) of the spoon, the functional part will be obstructed, rendering the intended task impossible to complete. Therefore, it is necessary to perform finer-grained, part-level segmentation to precisely locate functional grasping areas, such as the handle.

**Figure 4:**
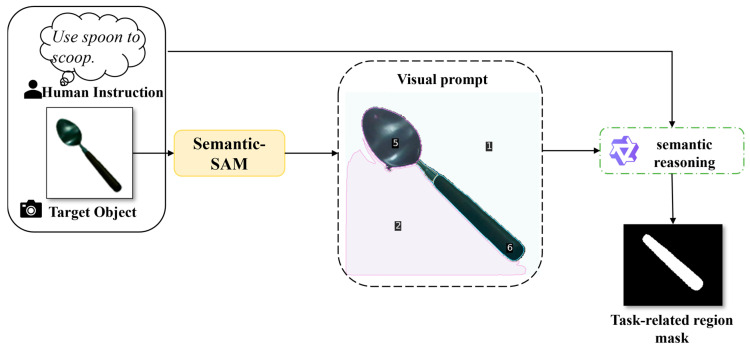
Fine-stage task-relevant part grounding framework. The numerical labels on the spoon indicate the indexed part-level segmentation results, which are used as visual prompts for VLM-based task-relevant region selection.

To this end, this study decouples the fine-stage part grounding process into a “Segment-then-Select” paradigm, as illustrated in Figure 4. First, inspired by the Set-of-Marks [25] approach, Semantic-SAM [26] is used to generate multiple part-level candidate regions within the target mask, each assigned a unique Visual Prompt Index. This design converts continuous pixel-level prediction into discrete region selection, simplifying task-relevant part localization.

**Figure 5:**
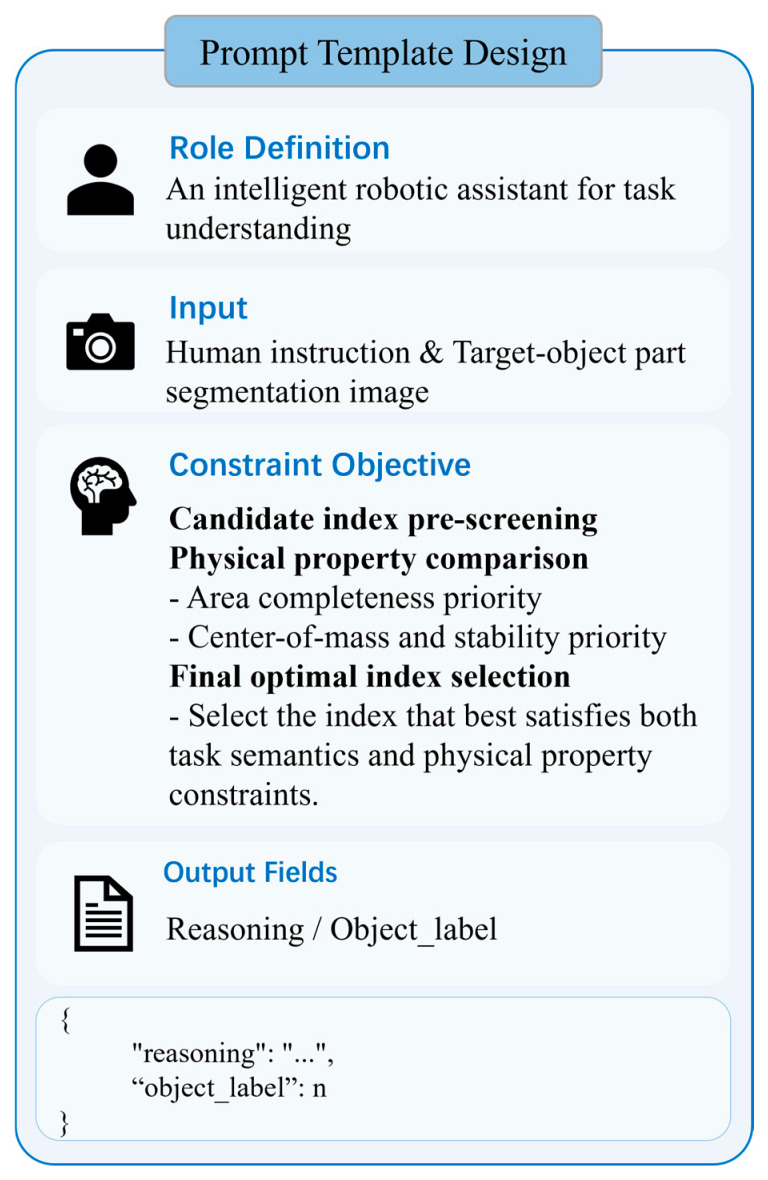
Prompt template for semantic reasoning between task instructions and candidate part regions in the fine stage.

In this process, the VLM serves as the core decision-making module. Instead of participating directly in low-level image segmentation, it performs logical alignment within the pre-generated pool of visual candidates by interpreting the task semantics of human instructions, precisely selecting the part index that best meets the task requirements. To achieve this goal, we have designed a specialized structured prompt template, as shown in Figure 5. By deeply fusing the visual index map with the task context, the template guides the model to output the final decision result. Finally, this decision is mapped back to the original image coordinate system, providing critical spatial constraints for subsequent grasp pose filtering.

### 2.3. Geometric Heuristic Part-Level Segmentation

**Figure 6:**
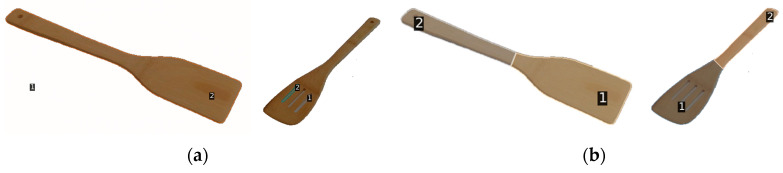
Demonstration of Semantic-SAM limitations on textureless and homogeneous objects. Subfigure (**a**) shows the segmentation result produced by Semantic-SAM, while subfigure (**b**) shows the result obtained using the proposed GHS method.

Although vision foundation models like Semantic-SAM excel in general part decomposition, they often struggle with textureless or surface-homogeneous objects commonly found in assistive scenarios, such as white plastic spoons or translucent containers. As illustrated in Figure 6a, these models frequently produce over-segmented or structurally incoherent regions due to the lack of distinct visual features.

To address this, we design GHS as a specialized geometry-based pathway. Instead of relying on visual semantics, GHS operates directly on the spatial distribution of the object’s binary mask. Specifically, the algorithm applies Principal Component Analysis (PCA) to the pixel coordinates to identify the principal axis of functional extension. Along this axis, the mask is partitioned into ***M*** equally spaced linear regions, where the granularity ***M*** is retrieved from the human experience database. This heuristic approach ensures that candidate regions remain aligned with the object’s physical structure, providing a reliable fallback when visual features are ambiguous. It is important to note that GHS is designed to complement, rather than replace, Semantic-SAM; the dynamic switching logic between these two pathways is detailed in Section 2.7. Figure 6b illustrates the segmentation results generated by GHS on the same objects.

### 2.4. Human-in-the-Loop Interactive Error Correction

While the automated segmentation and reasoning pipeline is efficient for routine grasping tasks, semantic alignment in the fine stage may still deviate when facing ambiguous instructions or highly complex unstructured environments (e.g., segmentation failure due to lack of visual features or semantic misinterpretation by the VLM). To enhance the decision-making robustness of assistive robots during complex manipulations, we introduce a HITL interactive error correction mechanism as a fail-safe, ensuring that the target operation area can still be accurately localized through human intervention when autonomous reasoning fails.

**Figure 7:**
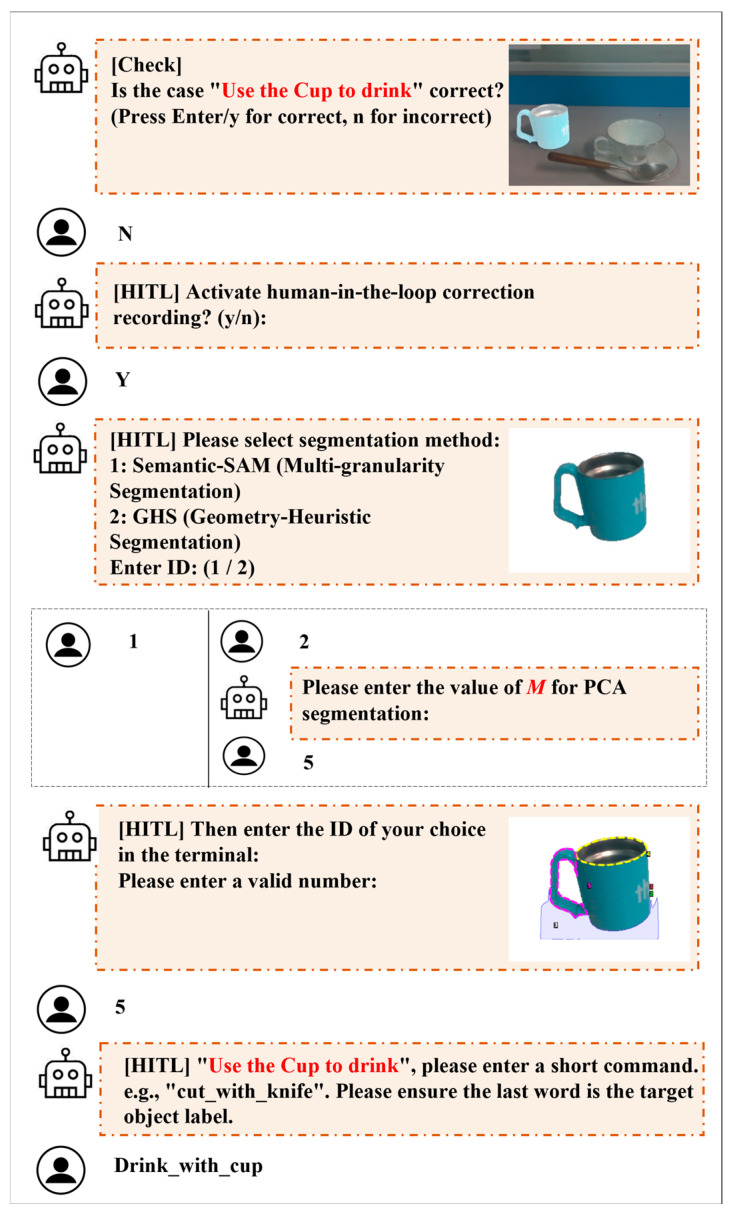
HITL correction process triggered when the automated pipeline fails. The robot avatar represents the proposed system, while the human avatar represents the user. Triggered by negative feedback (“N”), the user provides keyboard-based corrections, including error confirmation, segmentation method selection, PCA parameter *M* specification, final mask ID selection, and command correction.

The core logic of this module is to transform a complex autonomous reasoning process into high-confidence human verification and selection tasks. When the automated output of the fine stage does not meet expectations, the user can manually trigger the interactive mode. As illustrated in Figure 7, the interaction process primarily consists of two dimensions:

1. Strategy Selection: Based on the visual characteristics of the object (e.g., whether it is a typical textureless item), the user manually specifies the perception pathway (Semantic-SAM or GHS) to correct potential perception biases at the source.
2. Region Indexing: The system presents a part-level segmentation map annotated with numerical indices, allowing the user to provide the correct region index based on the task intent.

Through this streamlined interaction, the system effectively mitigates the randomness and uncertainty of VLM reasoning, precisely extracting the task-relevant region mask. Furthermore, it provides high-quality structured data for the subsequent construction of the human experience database.

### 2.5. Structured Human Experience Database Construction

To enable the system to evolve from single-task execution to long-term skill acquisition, the real-time correction data generated through HITL interactions must be transformed into structured, retrievable, and reusable experience exemplars for storage.

**Figure 8:**
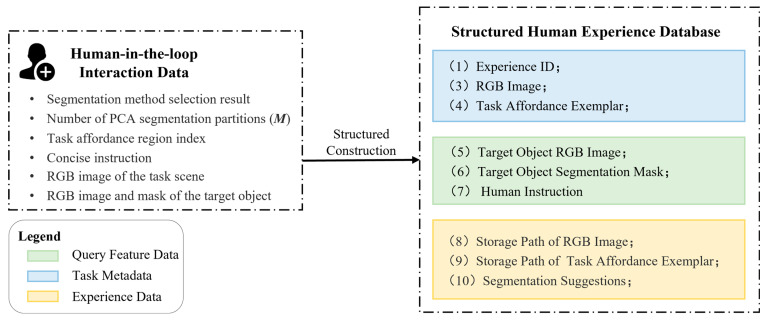
The transformation process from HITL interaction to structured information.

**Figure 9:**
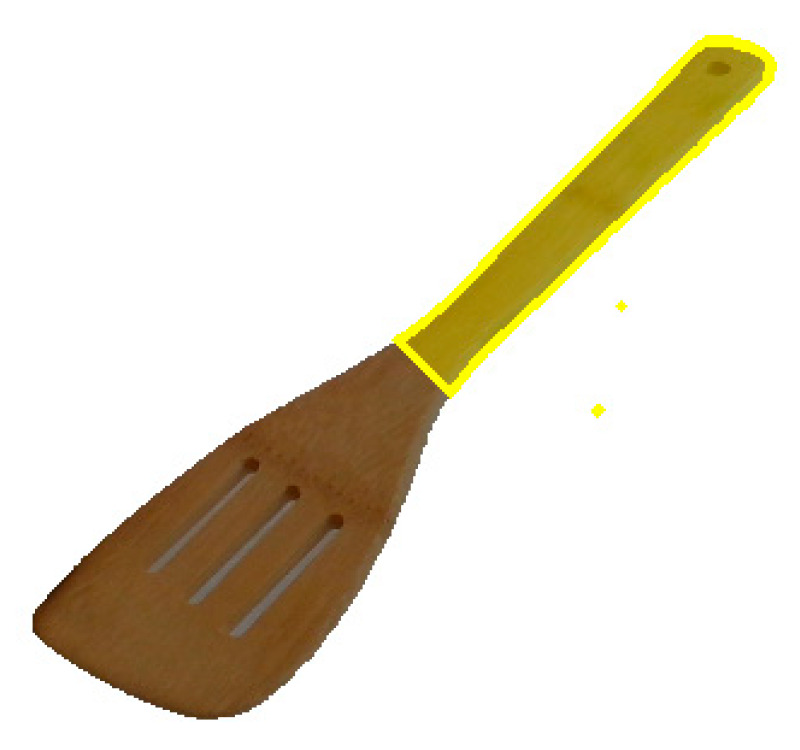
Illustration of task affordance exemplar.

The transformation process from HITL interaction to structured information is illustrated in Figure 8. During this stage, the system identifies and extracts the core elements of the interaction to define the information requirements for a complete “experience exemplar.” This specifically includes the following three categories of critical information:

1. Task Metadata: This component records the fundamental context of the task, including the experience ID, the raw RGB scene image, and the task affordance exemplar to ensure the traceability of environmental information. The affordance exemplar represents the task-relevant region mask output by the HITL process, as shown in Figure 9.

2. Query Feature Data: Designed to support subsequent multimodal experience retrieval, this part primarily consists of the human instruction corresponding to the task, the RGB image of the target object, and its associated segmentation mask.
3. Experience Data: This section records the core decision-making information derived from the HITL correction process, serving as the primary prior for future reasoning. Its content includes the storage paths for the scene image and the affordance exemplar, as well as segmentation suggestions. Among these, the segmentation suggestions primarily consist of the perception pathway identifier (i.e., Semantic-SAM or GHS) and the PCA partitioning granularity required by GHS. Notably, while the RGB scene images and affordance exemplars are categorized as Metadata, only their file paths are retained within the Experience Data component. This design not only aligns with the data input specifications of the VLM used in this paper (Qwen2.5-VL) but also effectively reduces memory overhead and transmission latency during frequent calls by utilizing path indexing instead of raw high-resolution image storage.

**Figure 10:**
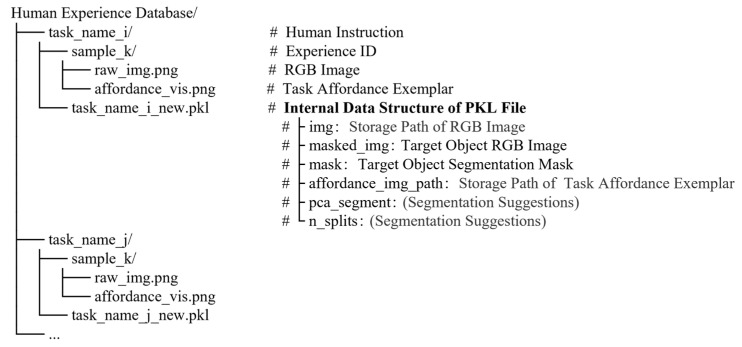
Hierarchical Task-Case storage architecture for the human experience database.

**Figure 11:**
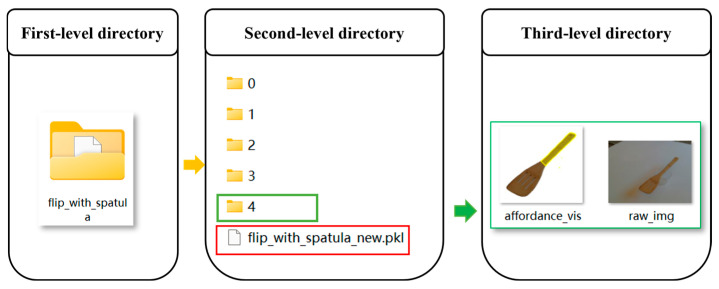
Example of structured storage for an experience exemplar in the human experience database. The experience database adopts a three-level directory structure, where the green box indicates the selected second-level folder and its third-level contents, and the red box highlights the corresponding .pkl file storing the structured experience information.

Once these information elements are defined, the system employs a hierarchical Task-Case architecture to physically organize and logically link the experience library, as shown in Figure 10. The knowledge base utilizes the human instruction (e.g., “scoop with a spoon”) as the primary root directory index, storing the transformed information through a combination of serialized files (.pkl) and image paths. This architecture ensures knowledge transparency while significantly enhancing the real-time inference efficiency of the system. As shown in Figure 11, an example of experience data storage in the human experience database is presented.

### 2.6. Multi-Level Experience Retrieval

To effectively leverage the prior knowledge stored in the human experience database constructed in Section 2.5 during downstream reasoning, we design a multi-level experience retrieval mechanism. The overall framework follows the hierarchical retrieval strategy in RAM, performing cascaded filtering across semantic, instance-level, and geometric dimensions. The detailed retrieval procedure is presented in Algorithm 1.
**Algorithm 1** Multi-level Experience Retrieval**Input**: Target Object Image $I_{q}$, Target Object Mask $M_{q}$, Task Instruction T, Target Object Label O, Structured Human Experience Database L **Output**: Exemplar $D^{*}$ **Stage 1: Task-Level Semantic Filtering**  1.Compute human instruction text embeddings ${\vec{e}}_{T}={\mathrm{CLIP}}_{text}(T)$  2.Compute text embeddings for each task $t_{i}$ in the Structured Human Experience Database L:
Compute ${\vec{e}}_{t_{i}}={\mathrm{CLIP}}_{text}(t_{i})$Compute cosine similarity $S_{LAN}(i)=\cos({\vec{e}}_{T},{\vec{e}}_{t_{i}})$
  3.Determine the set of candidate tasks $T_{cand}=\{t_{i}|S_{LAN}(i)\geq \tau_{LAN}\}$  4.Sort $T_{cand}$ in descending order of similarity $S_{LAN}$  5.Select the top-ranked task: $t^{*}=T_{cand}[1]$**Stage 2: Multimodal Instance Validation**  1.Initialize a global candidate list $R_{list}=\emptyset$  2.For the candidate task $t^{*}$:
Load demonstration data $D_{t^{*}}={\left\{({\mathrm{img}}_{j},{\mathrm{mask}}_{j})\right\}}_{j=1}^{N}$Visual-based CLIP filtering:
■Compute visual features ${\vec{v}}_{q}={\mathrm{CLIP}}_{vis}(I_{q})$, ${\left\{{\vec{v}}_{j}\right\}}_{j=1}^{N}={\mathrm{CLIP}}_{vis}({\mathrm{img}}_{j})$■Compute text features for the prompt “A picture of {O}”: ${\vec{e}}_{O}={\mathrm{CLIP}}_{text}(O)$■Compute joint similarity for each exemplar j: $S_{CLIP}(j)=\cos({\vec{v}}_{q},{\vec{v}}_{j})\times \cos({\vec{e}}_{O},{\vec{v}}_{j})$■Identify candidate exemplars: $P_{list}=\{D_{j}\in D_{t^{*}}|S_{CLIP}(j)\geq \tau_{CLIP}\}$

**Stage 3: Geometric refinement:**  1.For each exemplar $D_{j}\in P_{list}$:
Extract dense image features: ${\vec{f}}_{q}=\mathrm{DINOv2}(I_{q}⊙M_{q})$, ${\vec{f}}_{j}=\mathrm{DINOv2}({\mathrm{img}}_{j}⊙{\mathrm{mask}}_{j})$
  2.Compute Instance Matching Distance: $d_{j}={\mathrm{dist}}_{IMD}({\vec{f}}_{q},{\vec{f}}_{j},M_{q},{\mathrm{mask}}_{j})$  3.Final Exemplar Selection:
If $d_{j}\leq \tau_{IMD}$, then $R_{list}\leftarrow R_{list}\cup \{\left(D_{j},d_{j}\right)\}$Sort $R_{list}$ in ascending order of Instance Matching Distance $d_{j}$Select the optimal exemplar: $D^{*}=R_{list}[1]$Return $D^{*}$


Building upon this framework, and considering that our system prioritizes reliability over retrieval coverage, we introduce threshold-based conservative triggering conditions at each stage. Experience is activated only when sufficiently consistent matches are found; otherwise, the system falls back to the default reasoning pipeline, thereby avoiding the introduction of misleading priors in uncertain or out-of-distribution scenarios.

These thresholds are not designed for precise optimization of retrieval performance, but rather serve as constraint boundaries for experience activation. Their values are determined based on a preliminary analysis of similarity distributions within the human experience database, combined with sensitivity validation, to ensure stability and safety across different scenarios. The final output includes the selected task affordance exemplar, corresponding segmentation strategy, and reference visual information, which serve as priors for subsequent decision-making.

### 2.7. Retrieval-Driven Dynamic Reasoning Strategy

**Figure 12:**
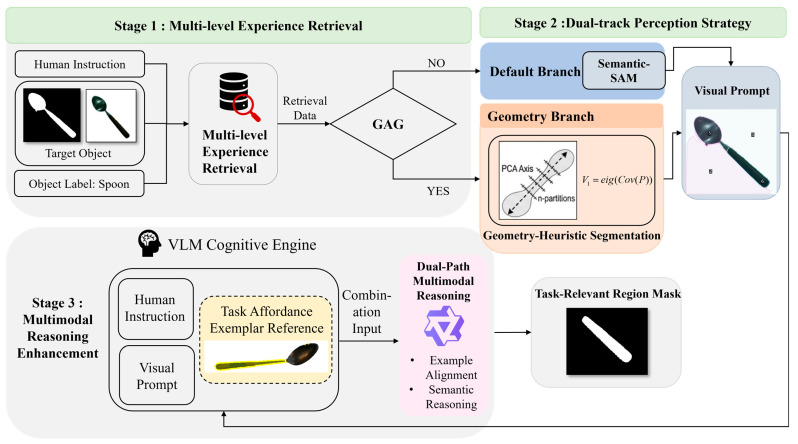
Retrieval-driven dynamic reasoning strategy framework. Based on the results of multi-level experience retrieval, the proposed strategy uses the Geometry Activation Gate (GAG) to dynamically schedule the dual-track perception strategy, selecting either the default Semantic-SAM branch or the GHS-based geometry branch. In the reasoning stage, the retrieval outcome further guides a dual-path reasoning strategy, switching between exemplar-alignment reasoning with retrieved task-affordance experience and default semantic reasoning. The numbers in the visual prompt denote indexed candidate part-level masks used as visual prompts for VLM-based task-relevant region selection rather than semantic category labels.

To achieve unified coordination among progressive segmentation, geometric heuristic segmentation, multi-level experience retrieval, and experience augmentation, we propose a retrieval-driven dynamic reasoning strategy. As illustrated in Figure 12, this strategy consists of two main components: a dual-track perception strategy and a dual-path reasoning strategy.

In the segmentation stage, the system dynamically selects the segmentation pathway via a Geometry Activation Gate (GAG). The GAG takes the retrieval results as input and jointly considers three conditions:

1. Whether task-relevant experience is retrieved;
2. Whether the retrieved exemplar adopts a geometric segmentation strategy (pca_segment = True);
3. Whether the Instance Matching Distance (IMD) between the current instance and the retrieved exemplar falls below a predefined threshold $\tau_{PCA}$.

Here, $\tau_{PCA}$ is used only to determine whether the GHS branch should be activated after a valid exemplar has been retrieved, and is different from the retrieval threshold $\tau_{IMD}$ used in the multi-level retrieval stage. GHS is activated only when all three conditions are satisfied. Otherwise, the system falls back to the default semantic segmentation branch (Semantic-SAM). Compared with fixed-rule or one-shot automatic triggering strategies, this work adopts an experience-driven branch scheduling strategy. Since household objects often reappear across scenarios, a single HITL correction can be stored as reusable experience to guide subsequent branch selection, balancing manual intervention cost, reasoning stability, and adaptability to diverse open environments.

In the reasoning stage, the system dynamically selects the reasoning pathway based on the retrieval outcome, forming a dual-path reasoning mechanism. When a high-confidence exemplar is successfully retrieved, the system adopts an exemplar-alignment reasoning mode; otherwise, it falls back to semantic reasoning based on the VLM. Notably, when the retrieved exemplar corresponds to geometric segmentation but fails to satisfy the GAG constraints, it is treated as invalid experience, and the system similarly reverts to the semantic reasoning pathway.

It should be noted that the retrieved exemplar does not directly modify the generated masks at the pixel level. Instead, the retrieval result first determines whether valid experience is available and what contextual information should be included in rag_context. Based on this, the system jointly considers the availability of valid experience, the segmentation strategy recorded in the retrieved suggestion, and the IMD constraint through GAG to select the segmentation pathway. When GAG is satisfied, GHS is activated, and the PCA partition granularity stored in the retrieved segmentation suggestion is used to control the number of geometric partitions. During reasoning, if valid experience is retrieved, the task affordance exemplar is incorporated into rag_context together with the current RGB input, converting the original semantic reasoning process into exemplar-alignment reasoning based on a previously verified correct case. If no valid experience is retrieved, the system falls back to the semantic reasoning pathway described in Section 2.2.

**Figure 13:**
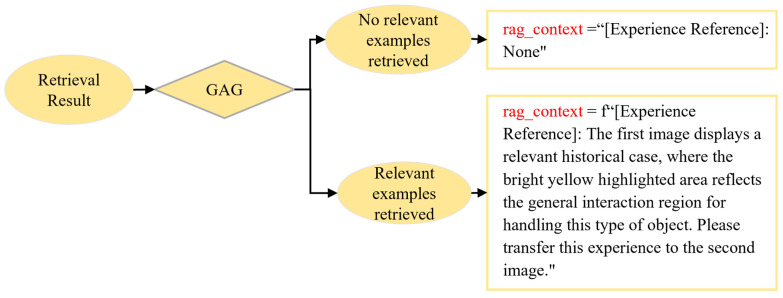
Construction process of prompt templates based on dynamic assembly strategy.

On this basis, we further design a dynamic prompt composition mechanism, which incorporates retrieved contextual information (rag_context) into the input prompt according to the availability of valid experience, as shown in Figure 13.

**Figure 14:**
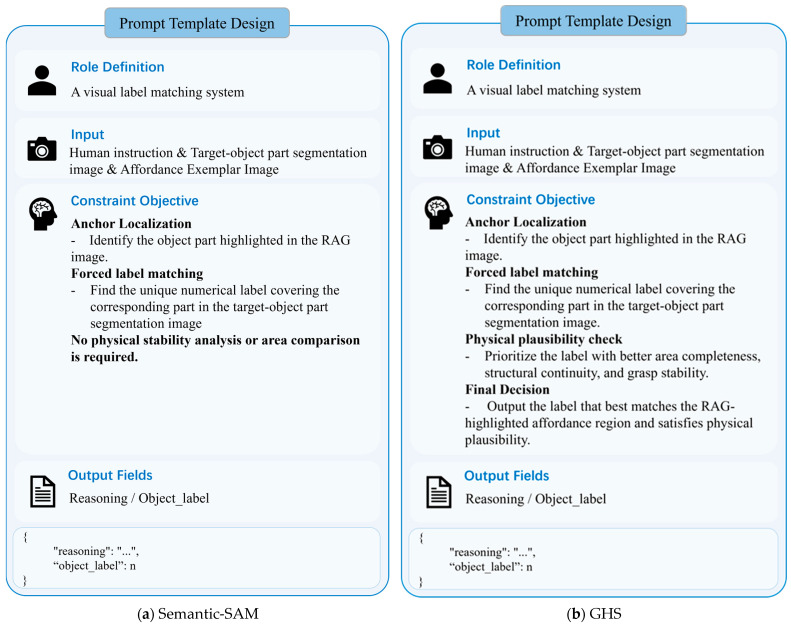
Exemplar-alignment reasoning prompt.

Considering that candidate regions generated by GHS are derived from geometric partitioning and may lack stable semantic correspondence, we design two types of prompts for exemplar-alignment reasoning: one based on Semantic-SAM candidates, emphasizing semantic consistency, as shown in Figure 14a; and the other based on GHS candidates, incorporating stronger structural and physical constraints to improve reasoning stability, as shown in Figure 14b.

### 2.8. Task-Oriented Grasp Pose Generation and Filtering

After obtaining the task-relevant region mask from the reasoning module, the system proceeds to the grasp pose generation and refinement stage. In this work, AnyGrasp [27] is adopted as the base grasp pose generator due to its strong zero-shot grasp detection capability in unstructured environments. As illustrated in Figure 1, the RGB image and depth image are first converted into a point cloud representation. Subsequently, AnyGrasp generates a set of global 6D grasp candidates in the scene, where each grasp is parameterized by a 6D transformation matrix, grasp width, and a confidence score.

To enable task-oriented manipulation, we perform task-oriented grasp pose filtering based on the inferred task-relevant region mask. This operator leverages the previously obtained task-relevant region mask to slice the global point cloud, retaining only the subset of points located within the task-relevant region (e.g., the handle of a spoon or a cup). Based on this constrained point cloud, the system performs hierarchical filtering over the candidate grasp set. First, all grasp candidates whose grasp centers lie outside the spatial bounding region defined by the task-relevant mask are discarded. Then, the remaining valid candidates are ranked according to the confidence scores predicted by AnyGrasp, and the optimal grasp pose is selected. Finally, the selected grasp is transformed into the robot base coordinate frame using predefined camera intrinsics and extrinsics.

By integrating general-purpose geometric grasping with fine-grained semantic constraints, the proposed approach ensures that the robot not only “grasps successfully,” but also “grasps appropriately” in accordance with the intended task.

## 3. Experiments and Results

### 3.1. Experimental Setup

**Figure 15:**
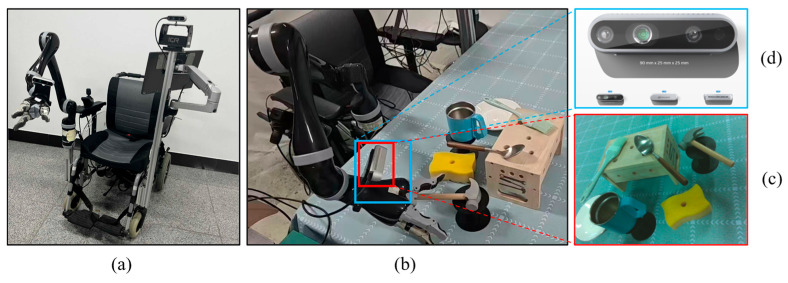
Overview of the WMRA platform and experimental setup. (**a**) Overall structure of the WMRA system, consisting of an electric wheelchair base and a Kinova Gen2 6-DOF three-finger robotic arm; (**b**) experimental setup of the WMRA platform; (**c**) representative experimental scenarios; (**d**) installation configuration of the Intel RealSense D435i depth camera.

The proposed method is experimentally validated on a wheelchair-mounted robotic arm (WMRA). The system is equipped with an embedded NVIDIA Jetson TX2 computing unit, which serves as the core processing module for algorithm execution. As illustrated in Figure 15a, the WMRA system integrates an electric wheelchair base with a Kinova Gen2 six-degree-of-freedom (6-DOF) three-finger robotic arm. For perception, the platform is equipped with an Intel RealSense D435i depth camera, and its installation configuration is shown in Figure 15d. The robot is developed based on the ROS framework, where the visual sensing system provides environmental perception, and the 6-DOF robotic arm enables precise object manipulation. The experimental setup and representative scenarios are shown in Figure 15b,c, respectively.

To comprehensively evaluate the performance of the proposed system, the experiments are divided into two parts: task-relevant region segmentation experiments on an evaluation dataset and real-world task-oriented grasping experiments in multi-object scenarios. For methods involving VLM, Qwen2.5-VL is adopted to ensure fair comparison.

Considering the limited diversity of objects in real-world experiments, we construct an offline evaluation dataset based on the TaskGrasp and Aff-Grasp [28] datasets, as detailed in Appendix A. The dataset covers 26 task categories, 39 object categories, and 104 object instances, resulting in a total of 247 task–object pairs. Each object instance contains multiple observations from different viewpoints, and a fixed viewpoint is selected in this work to form the evaluation dataset. On this dataset, we conduct performance comparisons between the baseline model and state-of-the-art methods. The baseline is defined as the original inference system without human experience enhancement, relying solely on pretrained VLM. In addition, for the HITL experience enhancement mechanism, we design sensitivity analyses of multi-level retrieval parameters and GHS thresholds $\tau_{PCA}$.

Before conducting the experience-enhanced experiments, we constructed the human experience database using a single offline round of HITL correction. Typical baseline failures were selected from two representative error types observed in the baseline results in Section 3.2.1: VLM semantic misunderstanding of task-relevant regions and segmentation failures caused by weak object textures. A total of 55 structured correction samples covering 16 task categories were collected. For each selected case, non-test-view observation images of the corresponding object instance were single corrected through HITL interaction and stored as structured experience exemplars. To avoid data leakage, no test-view annotations were included in the experience database. The constructed experience database was then fixed and used in the subsequent experience-enhanced experiments, including the sensitivity analysis of multi-level retrieval parameters in Section 3.2.2 and the sensitivity analysis of GHS thresholds in Section 3.2.3. The average time cost of each HITL correction was 12.81 s, mainly including user perception, decision-making, and region selection.

Real-world experiments are conducted in complex multi-object household scenes. For each task, a fixed set of 4–6 objects is selected to construct a cluttered scene with at least three object categories and background interference. For 8 representative task instructions, each method is executed 20 times under the same experimental protocol to reduce the influence of random factors and obtain statistically meaningful results. Across the 20 repeated trials of each method, the object categories remain unchanged, while their spatial poses and placements are randomly varied in each trial to ensure fair comparison.

To ensure fairness among different methods, all compared methods, including Baseline, Copa, AffordGrasp, and the original AnyGrasp, use the same grasp pose generation module based on AnyGrasp. The original AnyGrasp filters grasp candidates solely using object masks, while the other task-oriented methods further apply their predicted task-relevant region masks for grasp filtering.

**Figure 16:**
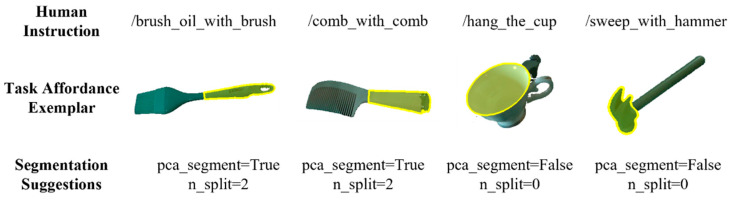
Representative human experience samples for complex affordance reasoning and textureless-object tasks.

The designed task set covers a range of difficulty levels, from semantically explicit tasks to more challenging affordance reasoning scenarios. Semantically explicit tasks, such as “Cut with the knife,” “Grasp the fork to poke,” “Use the spoon to scoop,” and “Grasp the hammer to beat nail,” usually involve relatively direct correspondences between the instruction and the functional grasping region. In contrast, more challenging tasks require implicit affordance reasoning. For example, tasks such as “Sweep the hammer across the table” require the robot to grasp the hammer head to enlarge the contact area, while “Hang the cup on the cup holder” requires grasping the cup body to release the handle for hanging. In addition, textureless-object tasks such as “Use a brush to brush some oil” and “Comb with a comb” are also included, where the task-relevant regions lack clear visual boundaries and therefore rely more heavily on semantic reasoning and experience-based guidance. To support these challenging cases, representative HITL correction samples are stored in the human experience database and reused by the proposed method during retrieval-augmented reasoning, as shown in Figure 16.

### 3.2. Task-Relevant Region Segmentation Experiments on the Evaluation Dataset

#### 3.2.1. Quantitative Comparison with Multiple Methods

This experiment aims to evaluate the effectiveness of the proposed coarse-to-fine reasoning framework for task-relevant region segmentation, as well as its generalization capability under complex semantic instructions and diverse object scenarios. The input consists of RGB image and human instruction, and the output is the task-relevant region mask of the target object.

For evaluation, the mean Intersection over Union (mIoU) is adopted to measure the consistency between the predicted masks and the ground truth annotations. Following common practice, a prediction is considered successful if the mIoU exceeds 0.5. The overall segmentation success rate is then computed as the proportion of successful predictions over all test samples. For each task–object pair, a single fixed-view image is selected for evaluation. The mIoU is computed on a per-sample basis and averaged over the entire test set.

For a fair comparison, all baseline methods follow their original inference pipelines as described in their respective papers, with a unified VLM (Qwen2.5-VL) adopted where applicable. Methods such as Copa and AffordGrasp inherently generate intermediate segmentation masks for grasp filtering, which are directly used as predicted task-relevant regions without modifying their core inference mechanisms. The segmentation success rate results are reported in Table 1.

From the overall results, the Baseline, which adopts the proposed coarse-to-fine two-stage framework without human experience enhancement, already achieves competitive segmentation performance. This demonstrates the effectiveness of the staged modeling strategy itself. By performing task-level semantic filtering and background suppression in the coarse stage, the system can focus visual attention on candidate regions of the target object, thereby reducing interference from complex backgrounds in subsequent reasoning.

The ablation study further shows that removing the coarse stage (w/o Coarse) leads to a substantial drop in performance to 53.04%, highlighting the critical role of the coarse stage in improving target object localization and suppressing irrelevant environmental interference.

Regarding the compared methods, Copa achieves a relatively low success rate of 26.32%, mainly due to its susceptibility to background interference during semantic alignment, which makes it difficult to reliably localize target regions in complex scenes. Although AffordGrasp incorporates affordance reasoning, its performance is limited by the generalization capability of the underlying visual perception model, making it less effective when handling unseen objects.

Despite the competitive performance of the coarse-to-fine baseline, several limitations remain. First, at the VLM reasoning level, the system may still exhibit deviations in understanding task-relevant regions when processing complex task instructions. Second, for objects with weak textures or highly uniform feature distributions, the lack of clear visual boundaries between affordance regions and non-task regions makes it difficult to achieve precise pixel-level segmentation.

In summary, the experimental results validate the effectiveness of the proposed coarse-to-fine two-stage framework in addressing complex semantic understanding and fine-grained spatial localization tasks.

#### 3.2.2. Sensitivity Analysis of Multi-Level Retrieval Parameters

This experiment aims to investigate the impact of threshold parameters in the multi-level retrieval mechanism on system performance, and to validate the effectiveness of the human experience enhancement mechanism in correcting semantic deviations in complex task understanding and segmentation failures for textureless objects. The human experience database used in this experiment follows the construction protocol described in Section 3.1. Based on this database, we evaluate how different retrieval threshold settings affect task-relevant region segmentation performance.

In the multi-level retrieval mechanism, three thresholds are used to filter candidate experience samples: language similarity threshold $\tau_{LAN}$, visual similarity threshold $\tau_{CLIP}$ and instance matching distance threshold $\tau_{IMD}$.

**Figure 17:**
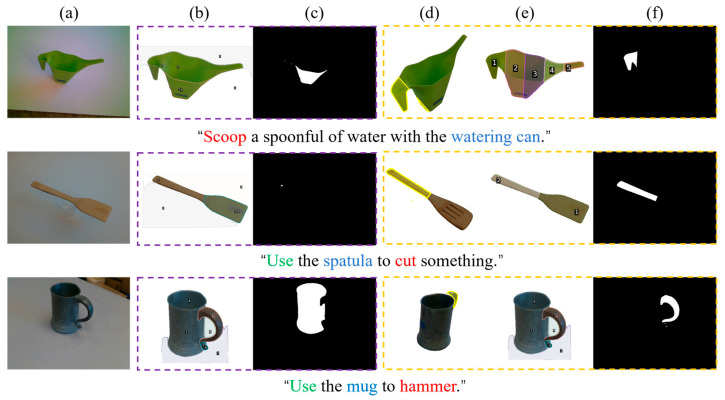
Qualitative comparison of task-relevant region predictions between the baseline and the proposed method with human experience enhancement. (**a**) Input RGB images. (**b**) Retrieved task affordance exemplars via multi-level experience retrieval. The results without human experience enhancement (baseline) are highlighted in purple, while those with human experience enhancement (proposed method) are highlighted in yellow. (**d**) and (**e**) Fine-stage segmentation results of the baseline and the proposed method, respectively. (**c**) and (**f**) Corresponding task-relevant region masks.

A total of 24 parameter combinations is evaluated on the dataset via exhaustive traversal, generating the corresponding task-relevant region masks. Qualitative comparisons between the baseline and the proposed method with human experience enhancement are presented in Figure 17, highlighting the effectiveness of experience-based correction in improving task-relevant region prediction. Notably, in Cases 1 and 2, the retrieved exemplars and the current inputs satisfy the activation conditions of the geometry branch, and the fine stage therefore performs segmentation using GHS.

To comprehensively evaluate system performance, in addition to the segmentation success rate (Table 2), mIoU is further adopted as a complementary metric (Table 3). While the success rate reflects the proportion of samples satisfying the threshold, mIoU provides a more fine-grained evaluation of the overlap quality between predicted regions and ground truth, enabling a more objective assessment of segmentation accuracy under different parameter settings.

The results show that, after introducing the experience enhancement mechanism, all configurations consistently outperform the baseline method (68.02%) in terms of segmentation success rate, indicating that the mechanism effectively improves overall system performance. Under the optimal parameter setting, the success rate reaches 74.09%, achieving an improvement of approximately 6 percentage points over the baseline. This demonstrates the effectiveness of experience-based knowledge in mitigating both VLM semantic reasoning deviations and segmentation failures caused by weak object textures.

Sensitivity analysis of the retrieval parameters further reveals that the system achieves better overall performance when $\tau_{CLIP}$ = 0.15, while performance slightly degrades when $\tau_{CLIP}$ = 0.2, indicating that overly strict visual similarity thresholds may suppress effective experience retrieval. For the instance matching distance $\tau_{IMD}$, the optimal range lies between 40 and 45, suggesting a good balance between retrieval effectiveness and geometric matching accuracy. Meanwhile, the setting $\tau_{LAN}$ = 0.7 yields the best overall trade-off under the selected visual and geometric thresholds, indicating that this threshold provides the most effective compensation for language reasoning. These results indicate that threshold settings must balance retrieval diversity and noise suppression.

Considering both segmentation success rate and mIoU, the optimal parameter combination is determined as $\tau_{LAN}$ = 0.7, $\tau_{CLIP}$ = 0.15, $\tau_{IMD}$ = 40 or 45, which yields the best overall inference performance.

#### 3.2.3. Sensitivity Analysis of GHS Thresholds

This experiment aims to investigate the impact of the activation threshold $\tau_{PCA}$ of the GHS mechanism on overall system performance, and to validate its necessity in handling textureless objects. The experiment is conducted based on the previously determined optimal retrieval parameter configuration ($\tau_{LAN}$ = 0.7, $\tau_{CLIP}$ = 0.15, $\tau_{IMD}$ = 40), while reusing the same human experience database described in Section 3.1. By varying the GHS activation threshold $\tau_{PCA}$ within the range of 0 to 40, we evaluate its impact on the dataset. During inference, the system takes the RGB image and human instruction as input, activates the experience enhancement module, and generates task-relevant region masks under different $\tau_{PCA}$ settings. The segmentation success rates are reported in Table 4. As the threshold increases, SSR generally shows an upward trend with slight fluctuations. When the threshold is set to 40, the SSR reaches the highest value of 77.73%, indicating that geometric priors can effectively improve task-relevant region localization.

When the GHS threshold is set to 0, the method only retains semantic-level retrieval enhancement and shows limited improvement over the baseline (68.42%), indicating that semantic alignment alone cannot reliably handle viewpoint-induced segmentation variability.

This phenomenon can be attributed to two main factors. First, cross-view variations may lead to unstable or fragmented masks generated by the segmentation model in the current image. Second, generative models inherently introduce uncertainty during semantic reasoning. By incorporating geometric priors from retrieved examples, GHS generates structurally regularized candidate regions, thereby improving the stability and accuracy of task-relevant region localization.

Overall, the experimental results highlight the importance of geometric priors in task-relevant region segmentation and further demonstrate the necessity of integrating semantic alignment with geometric structure guidance.

#### 3.2.4. Time Cost Analysis of HITL and Retrieval

To further evaluate the practical efficiency of the proposed system, we analyze the time cost of both HITL correction and retrieval-augmented reasoning. As described in Section 3.1, a total of 55 HITL interactions were conducted to construct the human experience database, resulting in 55 structured correction samples covering 16 task categories, with an average correction time of 12.81 s.

During inference, the retrieval process follows a multi-level retrieval strategy. When no similar task folder is found in the first-level retrieval, the system terminates early, resulting in an average time cost of 1.85 s. In cases where the full retrieval process is triggered, the average time cost is 5.09 s.

These results indicate that, although HITL introduces additional human interaction cost during the offline experience construction stage, the online inference process remains efficient. Moreover, the retrieval mechanism incurs only moderate computational overhead, especially considering that full retrieval is activated only under reliable matching conditions.

#### 3.2.5. Ablation Study Summary

To provide a clearer understanding of the contribution of each component, a summary of ablation results is presented in Table 5, where the coarse stage, retrieval module, and GHS mechanism are incrementally introduced.

The ablation results show that w/o Coarse obtains the lowest SSR of 53.04%, indicating that removing the coarse-stage target localization severely weakens the system’s ability to suppress background interference. w/o Experience improves the SSR to 68.02%, demonstrating the effectiveness of the basic coarse-to-fine pipeline. Compared with w/o Experience, w/o GHS achieves a slightly higher SSR of 68.42%, suggesting that semantic experience retrieval provides limited improvement when geometric refinement is absent. Finally, Ours-Full achieves the best SSR of 77.73%, showing that the combination of semantic experience retrieval and geometry-based experience with GHS provides complementary benefits for task-relevant region segmentation.

#### 3.2.6. Failure Case Analysis

**Figure 18:**
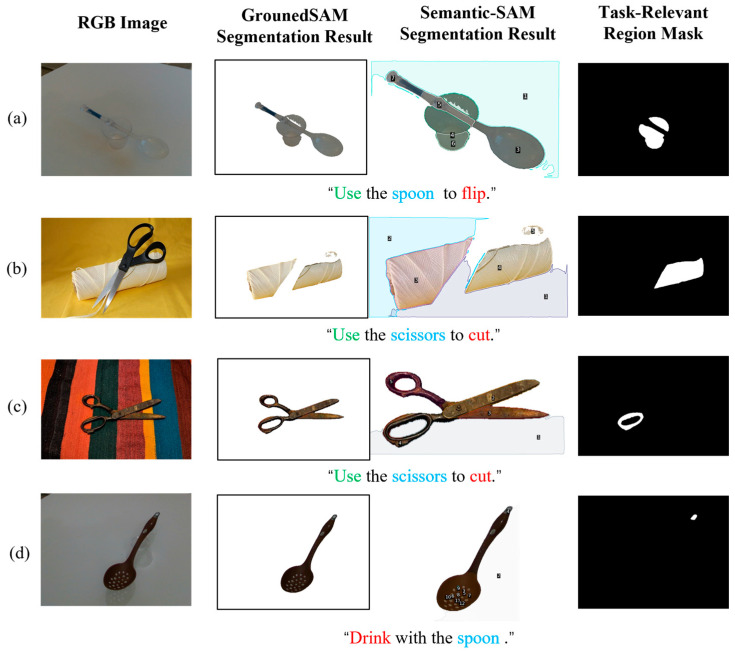
Representative failure cases of the proposed system in task-relevant region grounding. (**a**) Incorrect background selection caused by background regions being included in the fine-stage candidate masks; (**b**) coarse-stage target object localization failure, resulting in incorrect or incomplete fine-stage reasoning regions; (**c**) multiple plausible task-relevant regions, where the current system outputs only a single mask and may ignore other feasible manipulation regions; (**d**) unstable part-level candidates generated by Semantic-SAM, leading to inaccurate task-relevant region grounding.

To further analyze the limitations of the proposed system, several representative failure cases are shown in Figure 18.

Although the coarse-to-fine strategy with background suppression can significantly reduce background interference, several failure cases may still occur, as shown in Figure 18. These cases mainly involve incorrect background selection, coarse-stage localization failure, ambiguity caused by multiple plausible task-relevant regions, and unstable part-level candidate generation by Semantic-SAM. These observations indicate that the current system may still be affected by candidate mask quality, target localization accuracy, and the single-mask output strategy in complex real-world scenarios. In addition, although GHS is introduced to alleviate such segmentation failures, it is mainly suitable for objects with clear elongated or rod-like structures, and remains limited for objects with complex shapes or ambiguous geometric layouts. Furthermore, although the multi-level retrieval mechanism can usually reuse a single HITL experience in similar scenarios, effective retrieval may fail when the viewpoint variation is too large or when the current instance differs significantly from stored exemplars, in which case additional HITL correction is still required to enrich the experience base.

### 3.3. Real-World Experiments

#### 3.3.1. Robustness Analysis of GroundedSAM

To further evaluate the robustness of GroundedSAM under challenging real-world conditions, including occlusion, cluttered backgrounds, and varying illumination, we conduct qualitative experiments on four representative objects.

**Figure 19:**
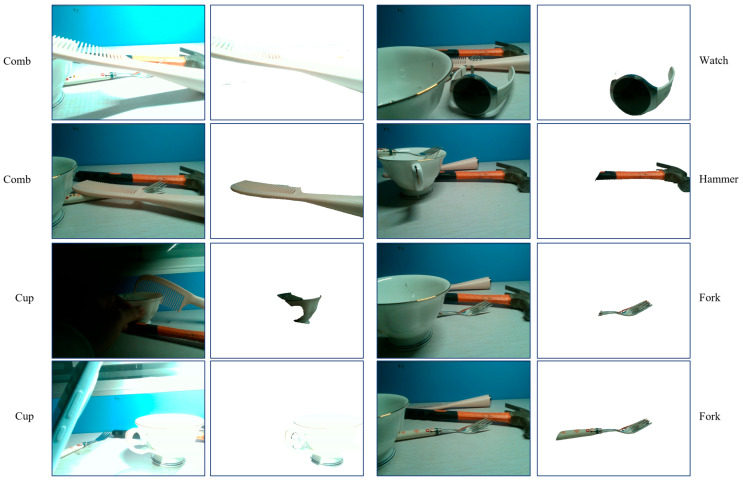
Qualitative segmentation results of GroundedSAM under occlusion, varying illumination, and complex backgrounds. Each pair shows the input RGB image with the target object label as the text prompt, and the corresponding target object segmentation result.

As shown in Figure 19, GroundedSAM demonstrates relatively stable detection performance across these scenarios. Although occasional false detections are observed, the method shows reasonable robustness in identifying target objects under moderate occlusion, complex backgrounds, and varying lighting conditions.

These observations suggest that GroundedSAM provides a reliable foundation for downstream affordance reasoning in diverse real-world environments.

#### 3.3.2. Real-World Task-Oriented Grasping in Multi-Object Scenarios

This experiment aims to evaluate the effectiveness of the proposed method in handling multi-object interference, viewpoint variations, and complex human instructions in real-world unstructured environments, with a focus on assessing the reliability of translating high-level semantic reasoning into low-level physical execution, particularly the improvement in grasp success rate brought by the human experience enhancement mechanism.

The closed-loop execution pipeline is as follows. First, the robotic arm moves to an initial observation pose, and RGB-D images are captured using an end-effector-mounted camera. The human instruction and the RGB image are then fed into the method to generate the task-relevant region mask. Next, AnyGrasp generates candidate grasp poses from the global point cloud, which are filtered by the predicted mask to retain only those within the task-relevant region. Finally, the optimal grasp is selected based on a quality score and executed by the robotic arm. A trial is considered successful if the robot grasps the target region and lifts the object without dropping it for 5 s.

In terms of target object segmentation success rate (Table 6), all methods except AffordGrasp demonstrate relatively stable object recognition across multiple tasks, indicating that current visual models have achieved strong robustness in object-level localization. This also shows that the main challenge in real-world task-oriented grasping does not lie only in locating the target object, but more critically in identifying the correct task-relevant region for manipulation.

**Figure 20:**
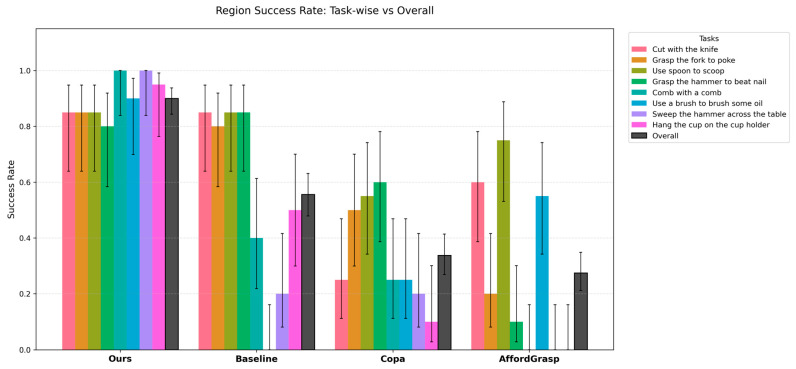
Task-wise and overall task-relevant region segmentation success rates with 95% confidence intervals across different methods.

In contrast, significant differences are observed in the success rate of task-relevant region segmentation. The task-wise and average segmentation success rates are summarized in Table 7, while the task-wise results with confidence intervals are further visualized in Figure 20.

For tasks with explicit semantics, such as “Cut with the knife”, “Grasp a fork to poke”, “Use the spoon to scoop” and “Grasp the hammer to beat nail”, the proposed method achieves performance comparable to the Baseline, indicating that the human experience enhancement mechanism is compatible with existing semantic reasoning and does not degrade performance on simple tasks. However, for tasks requiring more complex affordance reasoning or involving textureless objects, the proposed method achieves clearer advantages by retrieving relevant prior examples from the human experience database and using them to guide task-relevant region selection. Pairwise Fisher’s exact tests using Ours as the reference show that the overall improvements over the compared methods are statistically significant, with *p*-values lower than 0.05.

**Figure 21:**
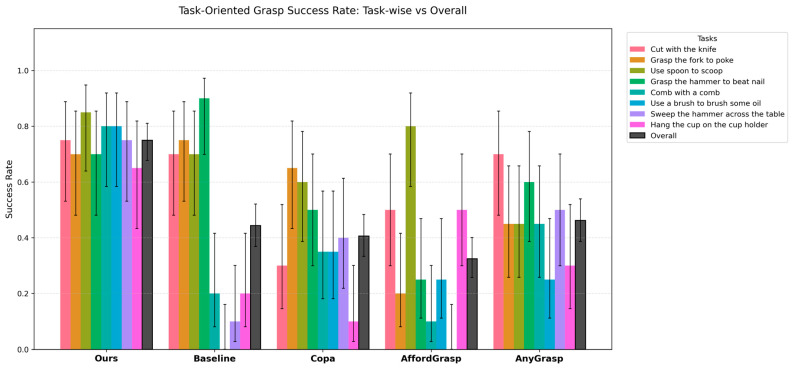
Task-wise and overall task-oriented grasp success rates with 95% confidence intervals across different methods.

Furthermore, the proposed method demonstrates clear advantages in the final grasp success rate. The task-wise and average grasp success rates are summarized in Table 8, while the task-wise results with confidence intervals are shown in Figure 21.

It should be noted that the comparison in Table 8 is not fully symmetric, since the full proposed method incorporates the HITL-enhanced experience database, whereas Copa, AffordGrasp, and AnyGrasp are evaluated without analogous human-supervised experience augmentation. The Baseline variant, which removes HITL enhancement while retaining the proposed coarse-to-fine pipeline, achieves an average grasp success rate of 44.375%, comparable to Copa (40.625%) and AnyGrasp (46.25%). Therefore, the improvement from 44.375% to 75% is mainly attributable to the HITL-enhanced experience augmentation, rather than the architectural pipeline alone. Experimental results show that the full proposed method achieves the highest average grasp success rate of 75%. Pairwise Fisher’s exact tests using the full proposed method as the reference further confirm that the overall improvements are statistically significant, with *p*-values lower than 0.05.

**Figure 22:**
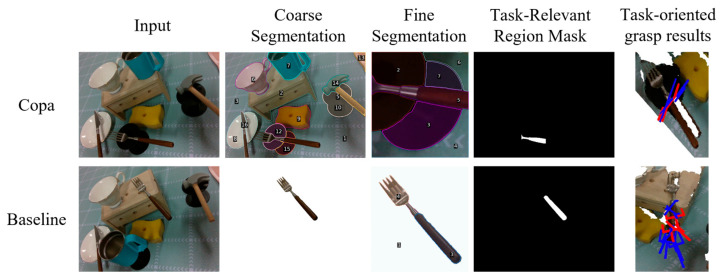
Comparison of valid grasp regions after task-relevant mask filtering.

Although Copa can perform task-relevant reasoning to some extent, it may produce incomplete task-relevant region masks due to the image cropping in its two-stage pipeline. This can limit the feasible grasp space after mask-based filtering, as illustrated in Figure 22. In contrast, the proposed method generates more complete and functionally consistent task-relevant regions, leading to more reliable task-oriented grasping performance.

**Figure 23:**
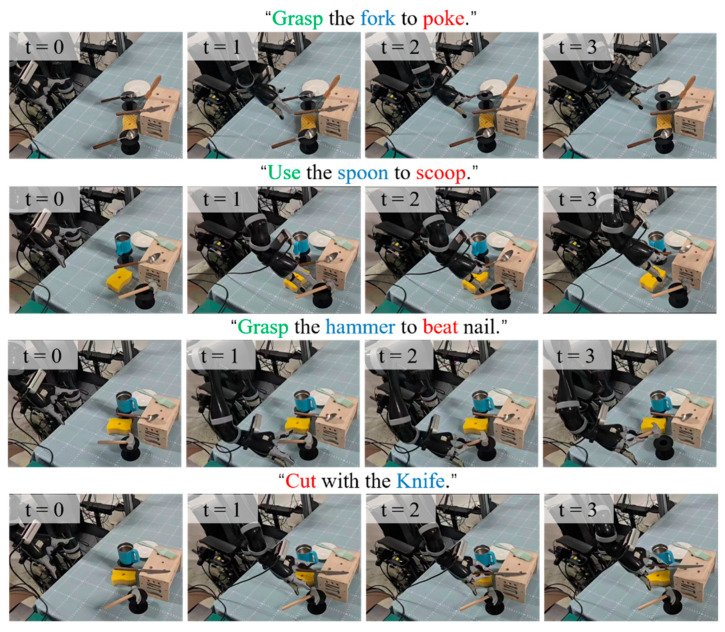
Representative real-world task-oriented grasping results of our method on simple tasks.

**Figure 24:**
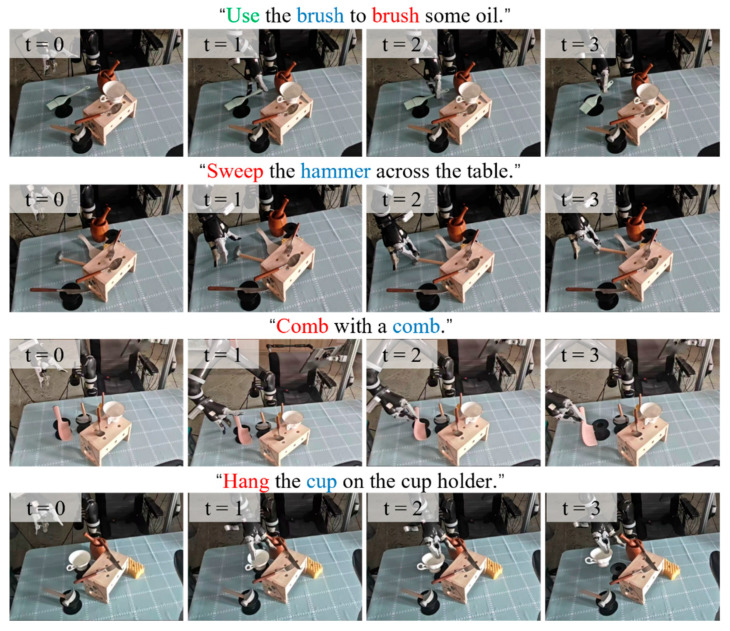
Representative real-world task-oriented grasping results of our method on more complex tasks, demonstrating its capability in handling challenging affordance reasoning scenarios.

To visually demonstrate the real-world execution process of the proposed system, representative task-oriented grasping trials on the real robot are shown in Figure 23 and Figure 24.

## 4. Discussion

This work systematically evaluates the proposed method from both quantitative and qualitative perspectives, demonstrating its effectiveness in bridging the gap between high-level semantic reasoning and low-level physical execution in task-oriented grasping. Compared with previous studies that primarily focus on geometric stability or predefined affordance representations, the experimental results show that integrating human experience into the perception pipeline significantly improves both segmentation accuracy and grasp success rate, particularly in complex scenarios requiring implicit affordance reasoning. These findings support the underlying hypothesis that experience-enhanced reasoning can effectively compensate for the limitations of VLM in fine-grained spatial understanding and functional inference.

From a broader perspective, the proposed framework highlights the importance of incorporating structured prior knowledge into embodied intelligence systems. By combining semantic reasoning, experience retrieval, and geometric consistency verification, the method provides a more reliable pathway for translating abstract task intentions into executable manipulation behaviors. This paradigm offers a promising direction for advancing robotic systems toward more generalizable and human-aligned decision-making in unstructured environments.

Despite these advantages, several limitations remain. The current grasping strategy focuses primarily on semantic localization and mask generation for task-relevant regions, without deeply integrating complex approach direction constraints at the perceptual level. Future research directions should focus on the end-to-end alignment of task-relevant action priors with 6-DOF pose generation models. This would enable the system to autonomously infer optimal manipulation configurations based on task intentions (e.g., lateral delivery or vertical placement), ultimately achieving refined manipulation that aligns more closely with physical common sense and human operational habits.

## Figures and Tables

**Table 1:** Segmentation success rate comparison of different methods.

Method	Segmentation Success Rate (%)
w/o Coarse	53.04
Baseline	**68.02**
Copa	26.32
AffordGrasp	47.77

Bold means the maximum value.

**Table 2:** Segmentation success rate (%) under different retrieval parameter settings.

$\tau_{IMD}$	$\tau_{CLIP}$ = 0.15			$\tau_{CLIP}$ = 0.2		
	$\tau_{LAN}$ = 0.6	$\tau_{LAN}$ = 0.7	$\tau_{LAN}$ = 0.8	$\tau_{LAN}$ = 0.6	$\tau_{LAN}$ = 0.7	$\tau_{LAN}$ = 0.8
35	72.47	70.04	72.06	69.64	72.06	72.87
40	72.06	73.68	71.26	70.04	70.85	71.66
45	**74.09**	**74.09**	71.66	70.04	72.87	69.23
50	73.28	71.26	72.87	70.45	69.23	72.47

Bold means the maximum value.

**Table 3:** mIoU under different retrieval parameter settings.

$\tau_{IMD}$	$\tau_{CLIP}$ = 0.15			$\tau_{CLIP}$ = 0.2		
	$\tau_{LAN}$ = 0.6	$\tau_{LAN}$ = 0.7	$\tau_{LAN}$ = 0.8	$\tau_{LAN}$ = 0.6	$\tau_{LAN}$ = 0.7	$\tau_{LAN}$ = 0.8
35	65.97	63.8	66	64.58	66.61	67.11
40	65.91	**67.74**	66.21	65.07	65.83	65.84
45	67.52	67.47	66.09	64.87	66.87	63.79
50	66.96	65.61	67.09	65.93	64.02	66.84

Bold means the maximum value.

**Table 4:** Segmentation success rate under different GHS threshold settings.

Method	Segmentation Success Rate (%)
Ours ($\tau_{PCA}$ = 0)	68.42
Ours ($\tau_{PCA}$ = 27)	73.28
Ours ($\tau_{PCA}$ = 29)	70.45
Ours ($\tau_{PCA}$ = 30)	73.68
Ours ($\tau_{PCA}$ = 32)	76.11
Ours ($\tau_{PCA}$ = 34)	72.87
Ours ($\tau_{PCA}$ = 36)	76.92
Ours ($\tau_{PCA}$ = 38)	76.92
Ours ($\tau_{PCA}$ = 40)	**77.73**

Bold means the maximum value.

**Table 5:** Ablation study summary of segmentation success rate.

Variant	Coarse	Fine	Exp (Semantic)	Exp (Geometry)	SSR (%)
w/o Coarse	-	✓	-	-	53.04
w/o Experience	✓	✓	-	-	68.02
w/o GHS	✓	✓	✓	-	68.42
Ours (Full)	✓	✓	✓	✓	**77.73**

Bold means the maximum value.

**Table 6:** Target object segmentation success rate (%) across different methods.

Human Instruction	Baseline	Ours	Copa	AffordGrasp
Cut with the knife	90	90	100	65
Grasp the fork to poke	100	100	90	20
Use the spoon to scoop	95	90	100	100
Grasp the hammer to beat nail	100	95	85	10
Comb with a comb	80	100	85	65
Use a brush to brush some oil	100	90	100	55
Sweep the hammer across the table	95	100	90	0
Hang the cup on the cup holder	95	95	95	60
Avg.	94.375	**95**	93.125	46.875

Bold means the maximum value.

**Table 7:** Task-wise and average task-relevant region segmentation success rate (%) across different methods.

Human Instruction	Baseline	Ours	Copa	AffordGrasp
Cut with the knife	85	85	25	60
Grasp the fork to poke	80	85	50	20
Use the spoon to scoop	85	85	55	75
Grasp the hammer to beat nail	85	80	60	10
Comb with a comb	40	100	25	0
Use a brush to brush some oil	0	90	25	55
Sweep the hammer across the table	20	100	20	0
Hang the cup on the cup holder	5	95	10	0
Avg.	50	**90**	33.75	27.50

Bold means the maximum value.

**Table 8:** Task-wise and average grasp success rate (%) across different methods.

Human Instruction	Baseline	Ours	Copa	AffordGrasp	AnyGrasp
Cut with the knife	70	75	30	50	70
Grasp the fork to poke	75	70	65	20	45
Use the spoon to scoop	70	85	60	80	45
Grasp the hammer to beat nail	90	70	50	25	60
Comb with a comb	20	80	35	10	45
Use a brush to brush some oil	0	80	35	25	25
Sweep the hammer across the table	10	75	40	0	50
Hang the cup on the cup holder	20	65	10	5	30
Avg.	44.375	**75**	40.625	26.875	46.25

Bold means the maximum value.

## Data Availability

The data presented in this study are available on request from the corresponding author due to project-related data confidentiality requirements.

## Abbreviations

The following abbreviations are used in this manuscript:

WMRA	Wheelchair-mounted robotic arm
HITL	Human-in-the-Loop
RAG	Retrieval-Augmented Generation
GHS	Geometric Heuristic Segmentation
LLM	Large Language Model
VLM	Vision-Language Model

## Appendix A

To evaluate task-relevant region segmentation, we construct a dataset for task-oriented grasping. The dataset is built by selecting task semantics and object instances from TaskGrasp, and further extended with additional object categories from Aff-Grasp to improve diversity and generalization. In addition, human instructions are automatically generated by Qwen 2.5 and subsequently verified and refined by human annotators to eliminate ambiguity, ensuring the accuracy and consistency of task descriptions.

The dataset consists of 10 task categories, 39 object categories, and 104 object instances, resulting in a total of 247 task–object pairs. It covers a wide range of everyday objects with diverse shapes and functional properties. The detailed dataset statistics are summarized in Table A1.

For annotation, we adopt the iSAT-SAM semi-automatic labeling tool. Two types of annotations are provided: goal_object for object-level localization and task_affordance for task-relevant operational regions. To reduce annotation complexity and improve consistency, all affordance regions are annotated using a single unified label without fine-grained categorization, ensuring annotation efficiency while maintaining reliable supervision for task-oriented region learning. This design also facilitates consistent training signals across different tasks, reducing ambiguity caused by overly fine-grained labels. Furthermore, by focusing on functional regions rather than category-specific attributes, the model achieves improved robustness in cross-task generalization, enhancing overall generalization, robustness, and stability. This formulation also aligns well with real-world task execution, where precise functional regions are more critical than fine-grained semantic distinctions.

**Table A1:** Dataset statistics across task categories.

Task	Object Categories	Instances
Cleaning	9	23
Cutting	6	23
Drinking	8	32
Flipping	4	25
Hammering	7	18
Scooping	16	58
Pouring	12	42
Stirring	8	36
Peeling	1	2
Brushing	1	2
